# A Retrospective Health Economic Analysis of a Stable Hypochlorous Acid Preserved Wound Cleanser Versus 0.9% Saline Solution as Instillation for Negative-Pressure Wound Therapy in Severe and Infected Wounds

**DOI:** 10.7759/cureus.24321

**Published:** 2022-04-20

**Authors:** Kathy E Gallagher, Emily C Alberto, Peter J Mallow, Michel H Hermans, Luis Cardenas

**Affiliations:** 1 Surgery/Acute Surgical Wound Service, ChristianaCare Health System, Newark, USA; 2 Surgery, ChristianaCare Health System, Newark, USA; 3 Health Economic and Clinical Outcomes Research, Xavier University School of Medicine, Cincinnati, USA; 4 Wound Care, Hermans Medical Consulting, Hoorn, NLD

**Keywords:** health economic analysis, wound management, complex wounds, npwt with instillation, negative pressure wound therapy

## Abstract

Introduction

Negative-pressure wound therapy (NPWT) with instillation and dwell time is an accepted adjunct therapy for infected wounds. A study was conducted to assess whether the use of hypochlorous acid preserved wound cleanser (HAPWOC) (Vashe, Urgo Medical North America, Fort Worth, TX, USA) as the irrigant would reduce the cost of care in comparison to 0.9% saline (NaCl).

Method

A comparative, observational, retrospective analysis assessed 27 serious and infected wounds in 24 patients. The lesions were of different and complex etiologies, including necrotizing fasciitis and stage IV diabetic foot ulcers. NPWT was used as part of the overall multimodal treatment regimen. The only variance in the treatment protocol was the use of saline (N=8) or HAPWOC (N=19) as the irrigant.

Results

When compared to NaCl, wounds treated with HAPWOC trended toward fewer operating room (OR) visits versus NaCl (3.3 versus 4.1) and a shorter length of hospital stay (LOS) (24.3 days versus 37.9 days).

The Orlando Health Transparency guide shows the cost of OR debridement as $2,525. Thus, debridement for HAPWOC-treated wounds ($8,332) costs $2,020 (24%) less than for NaCl-treated wounds ($10,352).

Using the 2016 Kaiser Health data (average daily hospital cost, excludingall interventions: $2,052), the cost of HAPWOC and NaCl instill translates to $49,864 and $77,771, respectively, a difference of $27,906 (56%) more for NaCl treatment. The Agency for Healthcare Research and Quality (AHRQ) 2012 data indicate an average daily cost of hospital stay, including all interventions, of $10,400. Thus, HAPWOC treatment cost translates to $252,720 versus NaCl-related costs of $394,160; in these calculations, using NaCl costs $141.440 (+56%) more per patient than HAPWOC.

Conclusion

The use of NPWT with HAPWOC versus NaCl as instillation in NPWT reduces the number of visits to the operating room and LOS. This has a significant impact on lowering the cost of care when HAPWOC is used.

## Introduction

The use of negative-pressure wound therapy (NPWT) was first described in the 1990s, and its usage has become the standard of care for several different indications [1-8]. NPWT is often combined with instillation therapy, and the combination of NPWT, instillation therapy, and dwell time (also known as contact time) has been shown to accelerate healing in certain types of wounds while decreasing wound bioburden [3,6,8-17]. NPWT combined with instillation may also reduce the burden of wound management on healthcare by decreasing visits to the operating room (OR) for debridement and shortening hospital length of stay (LOS) [3,18]. In our previous work, we demonstrated that the combination of NPWT with normal saline and sodium hypochlorite (Dakin’s solution, NaOCl) as instillation solutions with dwell time also increased the rate of formation of granulation tissue in severe and infected wounds [12].

Among the solutions used for NPWT instillation, a stable solution of 300 ppm hypochlorous acid preserved wound cleanser (HAPWOC) (Vashe, Urgo Medical North America, Fort Worth, TX, USA) has been shown to be noncytotoxic and nonirritating, with antimicrobial properties that make it safe for use tissues and shelf-stable for storage [19]. Given our success with Dakin’s solution as a wound irrigant, combined with our desire to switch to a less cytotoxic instillation solution, we began using HAPWOC in 2017 [12,19]. A prior study detailed our experience using HAPWOC compared to normal saline as NPWT instillation for severe and infected wounds [19]. The purpose of this study was to provide an economic analysis detailing the differences between HAPWOC and 0.9% sodium chloride (NaCl) as instillation in these wounds. A portion of this work was previously presented as audiovisual posters in May 2021 at the Symposium on Advanced Wound Care Spring Conference and the Nurses Specialized in Wound, Ostomy, and Continence Canada Conference.

## Materials and methods

The ChristianaCare Institutional Review Board (FWA00006557) approved a retrospective chart review of all patients with serious or infected wounds who were treated with NPWT and either HAPWOC or normal saline instillation between December 2015 and December 2017. In total, 24 patients with 27 wounds were eligible for inclusion in the study. The wounds were of multiple etiologies, including infected surgical wounds, traumatic injuries, deep and extensive pressure injuries, necrotizing fasciitis, fasciotomies, and vascular ulcers [19]. Major infections or necrosis were treated with aggressive surgical debridement before NPWT was initiated. NPWT was one component of a multimodal treatment program including nutrition optimization and systemic antibiotics.

Two solutions were used for instillation, HAPWOC and a 0.9% NaCl solution. The volume of solution used for each wound was determined by the wound size. Irrigant volume in milliliters was approximately 20% of the wound area in square centimeters. Wound depth was not included in the calculation for irrigant volume. The institutional protocol was used for the cyclic timing: dwell time was 10 minutes for every four hours of negative-pressure therapy, set at -125 mmHg. Dressings were changed every 2-3 days to allow for examination of the wound by the acute wound care team.

The data used in this analysis were published previously [12,19]. The collected data included patient demographics (e.g., age, sex, and comorbidities) and wound-specific data such as wound size, location, and etiology. Outcome data included time to wound closure (days), number of OR interventions, OR time, and LOS. In patients with more than one wound, each wound was separately evaluated for the aspects mentioned above, except for LOS, which was determined per patient. Patients with incomplete charts, those who were lost to follow-up, or those who died prior to wound closure were excluded from the analysis.

Data were summarized as follows: categorical variables were summarized with counts and percentages, while continuous variables were summarized with mean, median, standard deviation (SD), and range. Univariate analyses were performed using Student’s t-tests as previously described. Statistically significant differences were identified using a p-value set at 0.05.

The costs between HAPWOC and NaCl groups were estimated by multiplying the difference by published costs per unit. For instance, the price of OR time per cubic centimeter of wound can be calculated using the following equation [20]: average OR time spent multiplied by OR cost per minute divided by average wound size.

Similar calculations were made to estimate the cost of time to wound closure. The cost per OR intervention and LOS was estimated by multiplying the cost of a debridement procedure or inpatient day by the difference. All cost data were identified through a search of publicly available literature and assessed for relevance and recency by the authors.

## Results

Twenty-four patients with 27 wounds were included in the study (Table 1). In 17 patients with 19 (70%) wounds, HAPWOC was used as the irrigant, and in seven patients with eight (30%) wounds, 0.9% NaCl was used (Table 1). Three patients, two in the HAPWOC group and one in the NaCl group, had two wounds each, all secondary to fasciotomies for compartment syndrome. The average age of the patients was 49.7 and 36.1 years for the HAPWOC and NaCl groups, respectively (Table 1). In the HAPWOC group, eight (42.1%) wounds were related to trauma, and in the NaCl group, six (75%) wounds were traumatic [19].

**Table 1:** Demographic data

	HAPWOC	NaCl
Number of patients (male/female)	17 (13/4)	7 (6/1)
Average age (SD)	49.7 (15.1)	36.1 (19.3)
Number of wounds (%)	19 (70)	8 (30)

Fasciotomies were the most common wounds in both treatment groups, followed by other post-traumatic wounds. Infected surgical wounds were common in the HAPWOC cohort (Table 2). The lower extremities were the most common anatomical location of wounds for both treatment groups (Table 2). HAPWOC-treated wounds were larger than NaCl-treated wounds, with an average wound size of 304.6 cm^3^ and 174.9 cm^3^, respectively (p=0.08) (Table 2). The average number of comorbid diseases per patient was 3.3 in the HAPWOC group versus 1.7 in the NaCl group, with diabetes mellitus as the most common comorbidity. Intravenous drug abuse and the use of tobacco products also had a high prevalence (Table 3) [19].

**Table 2:** Wound data

	HAPWOC	NaCl
Number/percentage of total wounds	19	8
Fasciotomy	5 (26.3)	4 (50)
Post traumatic wound	4 (21.1)	2 (22.2)
Infected surgical wound	5 (26.3)	1 (11.1)
Necrotizing fasciitis	3 (15.8)	
Pressure ulcer	1 (5.3)	1 (11.1)
Vascular insufficiency ulcer	1 (5.3)	
Average size/SD (cm^3^)	304.6/292.9	174.9/174.1
Number/ percentage of total locations (%)		
Lower extremity	8 (42.1)	5 (62.5)
Upper extremity	2 (10.5)	1 (13)
Abdomen	5 (26.3)	1 (13)
Perineum/sacrum	4 (21.1)	1 (13)

**Table 3:** Main comorbidities and circumstances and number of patients

	HAPWOC	NaCl
Smoking	1	4
Intravenous drug abuse	2	1
Diabetes	4	1
Other comorbid conditions	4	0

Wounds treated with HAPWOC versus NaCl showed a trend toward fewer operative interventions (3.3 versus 4.1) and a shorter time to wound closure of 19.4 and 22.5 days for HAPWOC and NaCl, respectively (Table 4). Patients underwent instill therapy, on average, for 7.2 days in the HAPWOC group and 8.6 days in the NaCl group (Table 4). These differences did not reach statistical significance, however, because of the small number of patients in each group.

**Table 4:** Outcomes OR: operating room; SD: standard deviation; NPWT: negative-pressure wound therapy; LOS: length of stay

	HAPWOC	NaCl	Difference
Visits to OR, average (SD)	3.3 (2.3)	4.1 (2)	-0.8
NPWT with instill, days, average (SD)	7.2 (5.2)	8.6 (2.9)	-1.4
Time to wound closure (days), average (SD)	19.4 (9)	22.5 (18)	-3.1
LOS (days), average (SD)	24.3 (16.1)	37.9 (53.74)	-13.6
OR time/cm^3 ^(minute)	1.17	1.39 (19%)	-0.22
OR price/cm^3^ ($)	41.95	50.13 (19%)	-8.18

**Figure 1:**
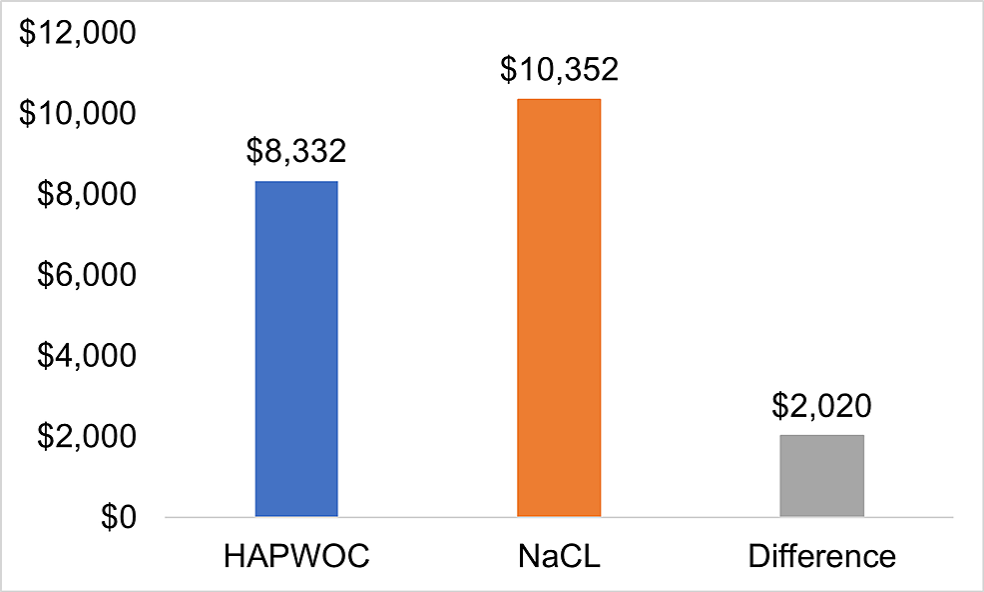
Cost of debridement HAPWOC: hypochlorous acid preserved wound cleanser; NaCl: 0.9% sodium chloride solution

In the United States, Current Procedural Terminology (CPT) codes are assigned for all medical interventions. Different CPT codes are used for debridement: the actual code depends on factors such as the size of the wound to be debrided, the depth, and the type of debridement. To standardize CPT usage for this study, we selected the lowest applicable CPT code for each debridement level. Medicare reimbursement for the simplest type of debridement (subcutaneous tissue including epidermis and dermis), CPT 11042, is $120 on average [21]. For NaCl-treated wounds, the minimum average price of debridement was $492, versus $396 for HAPWOC-treated wounds, representing a cost savings of $96 (24%) for HAPWOC-treated wounds. The Orlando Health Transparency guide shows the overall average price of debridement at $2,525 [22]. Thus, debridement of HAPWOC-treated wounds ($8,332) shows a cost savings of $2,020 compared to NaCl-treated wounds ($10,352), which indicates a similar overall cost savings of 24%, using this standard (Figure 1).

**Figure 2:**
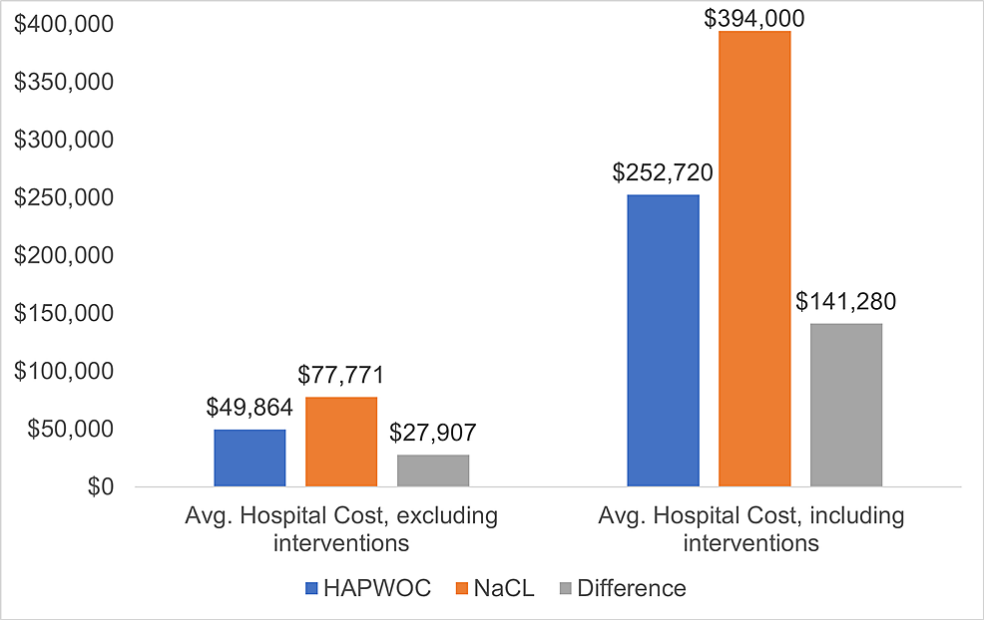
Average hospital cost HAPWOC: hypochlorous acid preserved wound cleanser; NaCl: 0.9% sodium chloride solution

The 2016 Kaiser Health data calculate the average daily hospital cost, excluding all interventions, at $2,052 [23]. This implies that the average cost of hospitalization, as determined by LOS, for patients treated with HAPWOC was $49,864. For patients treated with NaCl, the average cost was $77,771, a difference of $27,907 or 56% more for treatment with NaCl (Figure 2).

**Figure 3:**
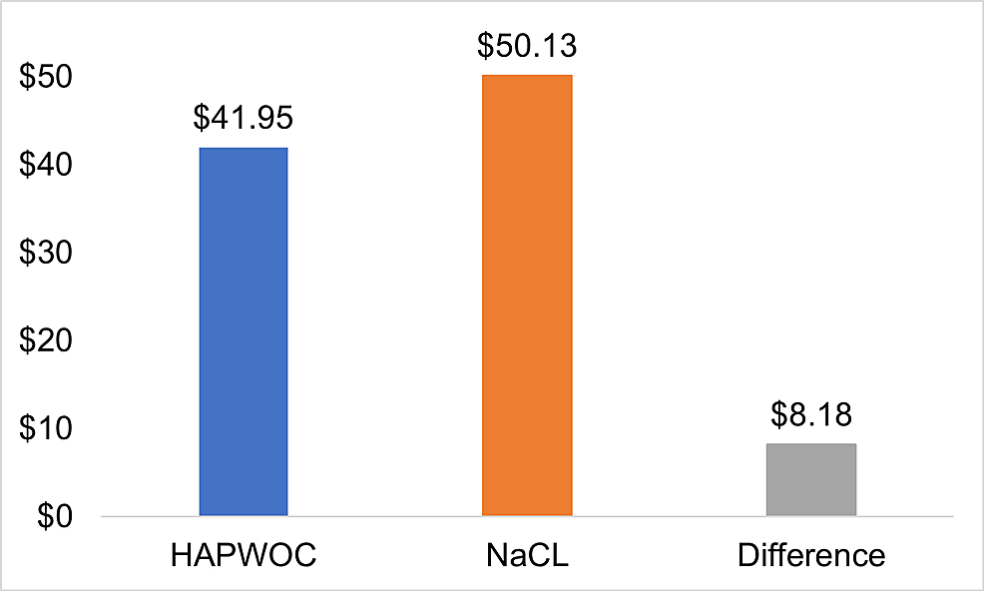
Operating room cost per wound volume (cm3) HAPWOC: hypochlorous acid preserved wound cleanser; NaCl: 0.9% sodium chloride solution

The Agency for Healthcare Research and Quality (AHRQ) 2012 data indicated an average daily price of hospital stay, including all interventions, of $10,400 [24]. Based on the LOS, using HAPWOC translates to an average of $252,720 per patient versus NaCl-related costs of $394,000. Calculating the incremental cost-efficiency ratio, using NaCl is $141,280 (56%) more expensive than using HAPWOC (Figure 2). Using financial data from California’s short-term general and specialty hospitals (2014 data), the mean price of OR time was estimated to be approximately $36 per minute [20]. The average number of minutes patients treated with NaCl spent in the OR was 242 (165-529) minutes, while the average OR time of patients with wounds treated with HAPWOC was 354 (range: 0-887) minutes. Therefore, time spent in the OR (average wound size divided by average time spent) is 1.39 and 1.17 minutes per cubic centimeter of wound volume for NaCl and HAPWOC, respectively. For the wounds treated with NaCl, this equates to an average OR cost of $50.13/cm^3^, and for HAPWOC, this number is $41.95/cm^3^, which represents a reduction of OR cost of $8.18/cm^3^ of wound in favor of HAPWOC over NaCl or a 19% price increase for the wounds treated with NaCl instillation (Figure 3).

## Discussion

Through retrospective chart review, we identified patients with serious and complex wounds who, as part of overall treatment including surgical debridement, were subsequently treated with NPWT combined with instillation and dwell time. The instill irrigants used were HAPWOC or 0.9% saline. The wounds had different etiologies, which reflect the multifaceted patient population served on the trauma-affiliated acute surgical care wound service. Patients in the HAPWOC cohort were older on average and suffered from more comorbidities and larger wounds. Patients in this group also had a higher rate of diabetes mellitus, which is known to be a detriment to wound healing [25-27].

NPWT has become the standard treatment for several different indications [3,5-7]. For wounds in diabetic patients, NPWT is known to increase granulation tissue and wound vessel density, which indicates an increase in healing potential [12,28]. The combination of NPWT with instillation and dwell time was demonstrated to further accelerate healing in certain types of wounds and decrease wound bioburden [3,6,14,17]. The purpose of the study described here was to evaluate whether the use of HAPWOC as the irrigant would improve clinical results (i.e., faster complete reepithelialization) when compared to 0.9% saline (NaCl) and whether there would be health economic consequences, as previously demonstrated [19].

In the previous study, we did find a shorter LOS, fewer operative interventions, and a faster time to healing for patients treated with HAPWOC [19]. In addition, the price of the surgical interventions differed significantly between the two instillation options, with HAPWOC offering a 19% ($8.18) price reduction per cubic centimeter of wound volume when considering the cost per minute of the OR time. The OR time spent per cubic centimeter of wound was also reduced from 1.39 to 1.17 minutes (19%) per unit of wound volume. While modern anesthesia does not report serious side effects or complications, a reduction of anesthesia time is in the best interest of the patient [29,30].

HAPWOC instillation was also favorable for other health economics-related prices, such as a reduced length of stay and overall estimated prices of care. Whether only hospital prices or hospital prices including interventions are analyzed, treatment with HAPWOC offered a price reduction of 56% (Figure 1).

Limitations

Several limitations are implicit in this type of study. The small number of patients who participated in the study limits the statistical power, although we showed clear trends in favor of HAPWOC treatment. Confirmation of these findings could be investigated in a large multicenter setting. The heterogeneity of the patient population and wounds, while a representative of the institution, complicate the assessment: comorbidities and the different wound etiologies themselves play a key role in all aspects of the healing process. Further, we did not have access to patient-specific cost data and relied on calculated estimates using published national data. Thus, the cost estimates are not necessarily reflective of our institution. However, they may be more generalizable to a nationwide population. To standardize measures of cost between patients included in the study, we used the lowest level of CPT debridement reimbursement. Lastly, we used our own wound treatment protocol that may not be representative of the treatment of similar wounds in other institutions and clinical settings.

## Conclusions

Patients with complex wounds underwent treatment with NPWT with two types of instillation therapy, a stable hypochlorous acid preserved solution and 0.9% saline, as part of their overall treatment regimen. Although the HAPWOC-treated wounds were larger and patients were older with more comorbidities, our studies identified fewer OR visits, faster wound closure, and decreased length of stay for HAPWOC when compared to normal saline as the negative-pressure instillation fluid. Using literature-based cost data, the total price of treatment was 56% lower in the HAPWOC cohort when compared to NaCl-treated wounds. The price of the OR time per cubic centimeter of wound for HAPWOC was 19% lower than for NaCl-treated patients. This represents a difference of $8.18 per cubic centimeter of wound volume in favor of HAPWOC treatment. From an economic perspective, the use of HAPWOC leads to an impressive reduction of overall costs of treatment. Although we were unable to show statistical significance at the chosen primary endpoints, due to our low number of study patients, we do feel this warrants further consideration and clinical trials.
